# Patients with type 1 diabetes mellitus and recommended low-density lipoprotein cholesterol, non-high-density lipoprotein cholesterol and apolipoprotein B targets – a SwissDiab study

**DOI:** 10.3389/fendo.2026.1819019

**Published:** 2026-05-29

**Authors:** Christina Liu, Frida Renström, Giacomo Gastaldi, Michael Brändle, Stefan Bilz

**Affiliations:** 1 Division of Endocrinology and Diabetes, Cantonal Hospital St.Gallen, HOCH Health Ostschweiz, St. Gallen, Switzerland; 2 DiaCentre – Maison Du Diabète, Hirslanden Hill Clinic, Chêne-Bougeries, Switzerland; 3 Department of Medical Specializations, Diabetology, Geneva University Hospitals, Geneva, Switzerland; 4 Division of General Internal, Family, and Emergency Medicine, Cantonal Hospital St. Gallen, HOCH Health Ostschweiz, St. Gallen, Switzerland

**Keywords:** agreement in lipid target attainment, apoB, cardiovascular risk, LDL-cholesterol, lipid management, non-HDL-cholesterol, type 1 diabetes mellitus

## Abstract

**Background:**

Cardiovascular risk in type 1 diabetes mellitus (T1DM) is frequently underestimated in daily clinical practice. The aim was therefore to evaluate lipid management in patients with T1DM according to current guidelines, and agreement in target attainment across three recommended lipid biomarkers.

**Methods:**

Target attainment of (low-density lipoprotein cholesterol [LDL-C, estimated with the Friedewald formula], non-high-density lipoprotein cholesterol [non-HDL-C], and apolipoprotein B [apoB]) was determined based on the 2021 European Society of Cardiology Dyslipidemia guideline among adult patients with T1DM enrolled in SwissDiab.

**Results:**

Median age of the 216 patients included was 43.6 years, diabetes duration 16 years, HbA1c 7.3% (56 mmol/mol), 39.4% were females, 75.0% were at high, and 11.6% at very high cardiovascular risk. Prescription of lipid-lowering therapy (LLT) was low (33.6%). LDL-C target attainment was poor (11.6%), while significantly more patients reached non-HDL-C (30.6%) and apoB (59.7%) targets (P-values <0.00001). Overall, 11.1% of the patients reached all three, and 39.8% none of the lipid targets.

**Conclusion:**

Raised awareness of the importance of lipid management in T1DM is needed. However, the observed discordance in target attainment across lipid biomarkers can lead to uncertainties in clinical decision-making on how to best apply current lipid management recommendations for optimal cardiovascular risk prevention in T1DM.

## 1 Introduction

Due to premature atherosclerotic cardiovascular events, type 1 diabetes mellitus (T1DM) is associated with an almost threefold higher mortality and 13 years shorter lifespan compared to the general population (1–3). The mechanisms underlying atherosclerotic cardiovascular disease (ASCVD) in T1DM, although in large parts overlapping, may differ from that observed in type 2 diabetes mellitus (T2DM) (1, 4). Traditional cardiovascular risk factors aside, early disease onset, poor glycemic control, and prolonged exposure to hyperglycemia may contribute to the increased ASCVD risk in T1DM (5). Data indicate that more than 70% of men and 50% of women with T1DM have developed coronary artery calcifications by the age of 45 (6). A recent publication showed a higher risk of heart failure and chronic kidney disease among patients with T1DM across ages, a higher risk of myocardial infarction starting middle age, and a higher risk of stroke at younger ages compared to patients with T2DM (7). Although tight glycemic control improves cardiovascular outcomes (8), data from the Swedish diabetes registry show a twofold increased risk in patients with well-controlled T1DM compared to the general population (2).

One of the key modifiable drivers of ASCVD is hyperlipidemia. Low-density lipoprotein cholesterol (LDL-C), non-high-density lipoprotein cholesterol (non-HDL-C) and apolipoprotein B (apoB) are three key biomarkers that have been shown to independently predict cardiovascular outcomes in various populations (9–12). Non-HDL-C accounts for the risk conferred by both triglyceride rich lipoproteins and LDL-C and has consistently been shown to enhance ASCVD risk prediction in T2DM when compared to LDL-C. Therefore, and due to its wide availability, it is recommended for ASCVD risk prediction in current European guidelines for patients with and without T2DM (13, 14). Plasma apoB concentration reflects the number of all circulating atherogenic lipoproteins and has been identified as an even more accurate lipid surrogate for ASCVD risk when compared to LDL-C and non-HDL-C (10, 15).

An overwhelming body of evidence indicates that the cumulative exposure to atherogenic lipoproteins determines lipid-attributable ASCVD risk and the risk reduction conferred by lipid-lowering therapy (LLT) is dependent on the absolute magnitude of LDL-C lowering (16, 17). Although no randomized cardiovascular outcome trials have investigated the effect of LLT in T1DM, data from the Swedish Diabetes Registry show lower all-cause mortality (~44%), risk of stroke (~44%), and fatal and non-fatal myocardial infarction (~25%) in patients with T1DM treated with statins (18). Following these findings, recent European guidelines unequivocally advocate early and intensive lipid lowering in patients at high or very high ASCVD risk, among them many with T1DM (19–22), and the recent European Society of Cardiology (ESC) guidelines regarding lipid management, as delineated in **Table 1**, now include recommended targets for LDL-C, non-HDL-C and apoB (20, 21).

**Table 1:** Recommended lipid targets stratified by atherosclerotic cardiovascular risk according to the 2021 guidelines on cardiovascular disease prevention in clinical practice by the European Society of Cardiology (21).

ASCVD risk	LDL-cholesterol	Non-HDL-C	ApoB
Moderate	<2.6 mmol/L	<3.4 mmol/L	<1 g/L
High	<1.8 mmol/L and ≥50% reduction from baseline	<2.6 mmol/L	<0.8 g/L
Very high	<1.4 mmol/L and ≥50% reduction from baseline	<2.2 mmol/L	<0.65 g/L

However, discordance between LDL-C, non-HDL-C, and apoB levels has been observed in population-based studies (23, 24), the INTERHEART study, a standardized case-control study of acute myocardial infarction (25), as well as in T2DM (26, 27). Information on discordance in T1DM is more limited, but has been observed between LDL-C and non-HDL-C (27), LDL-C and apoB (28), and non-HDL-C and apoB (29). With the current recommendations, the lipid management strategy pursued in cases of discordance is primarily at the discretion of the attending physician. To what extent this might constitute a gap in our knowledge and treatment of dyslipidemia in T1DM remains largely unclear. The aim of the study was therefore to assess the proportion of patients with T1DM in the Swiss Diabetes Registry (SwissDiab) that reach the lipid targets recommended by ESC 2021 (21), and the agreement in target attainment across the three guideline-recommended lipid biomarkers (LDL-C, non-HDL-C, and apoB).

## 2 Materials and methods

### 2.1 Study participants

SwissDiab is a multicenter longitudinal observational study of outpatients in tertiary diabetes care at HOCH Health Ostschweiz, Cantonal Hospital of St. Gallen (coordinating center), and Basel, Bern, Geneva and Zürich University Hospital. The overall aim is to provide feedback on daily clinical practice by assessing diabetes care and management, prevalence and incidence of diabetes-related complications, and quality of life. Patients ≥18 years of age are eligible for participation, regardless of diabetes type (gestational diabetes excluded), duration, or treatment. Patients with irregular attendance (for instance due to drug abuse or mental disorder) or a life expectancy <1 year due to severe comorbidity are excluded. Diabetes is diagnosed clinically according to the American Diabetes Association, supported by autoantibody assessment when appropriate. Participating patients attend a standardized annual health examination where medical history, diabetes-related complications, cardiovascular risk factors, biochemistry, and current medication is collected by trained medical staff. Written informed consent was provided by all SwissDiab participants, and the study protocol was approved by the Ethics Committee East Switzerland (EKOS) and the additional local ethics committees (BASEC-ID PB_2016-01449).

### 2.2 Study design

Eligible for participation was patients with T1DM and a SwissDiab visit between 01.01.2021 and 09.01.2023 with a complete set of data on LDL-C, non-HDL-C, and apoB. The most recent visit was used, unless missing data justified the inclusion of a previous visit. Cardiovascular risk and corresponding lipid targets were determined according to ESC 2021 (21). Patients with a diabetes duration <10 years without target organ damage or cardiovascular risk factors were considered at moderate risk. Patients with established ASCVD and/or severe target organ damage, or LDL-C >5 mmol/L were considered at very high risk. Patients that were not classified at either moderate or very high risk were considered at high risk (**Supplementary Table 1**).

For patients on LLT, treatment naïve LDL-C levels were extrapolated based on current LLT in accordance with the 2019 ESC/EAS guidelines for the management of dyslipidemias (20). Based on reported average dose-response reductions, the following treatment combinations were considered to achieve ≥50% reduction of baseline LDL-C; moderate intensity statin in combination with ezetimibe (50-65%), and any treatment including high intensity statin (>50%) or proprotein convertase subtilisin/kexin type 9 inhibitor (PCSK9i, >60%) (20, 30). If information on LLT was missing but LDL-C was above the recommended target, patients were defined as not having reached target.

### 2.3 Clinical characteristics

Weight was measured with a digital scale with patients wearing light clothes without shoes. Height was measured with a wall-mounted stadiometer. BMI was calculated as weight (kg) divided by height (m) squared. Systolic and diastolic blood pressure was measured following a 5-minute rest with the patient in a seated position. In case blood pressure was measured on both arms, the higher finding was used. Arterial hypertension was defined as blood pressure levels above 140 mmHg systolic and 90 mmHg diastolic and/or treatment with antihypertensive medication.

### 2.4 Biochemistry

Patients were advised to arrive fasted (>8 hours). Serum TG and total cholesterol (TC), HDL-C, and LDL-C levels were determined using enzymatic colorimetric tests according to routine methods at the center of laboratory medicine at each hospital. Based on directly measured TC, TG and HDL-C, LDL-C was calculated based on the Friedewald formula as TC – HDL-C – (TG/[2.2]) (31). Analysis was repeated with LDL-C estimated by Sampson (TC/0.948 − HDL-C/0.971 − (TG/8.56 + [TG × non-HDL-C]/2140 − TG^2^/16100) − 9.44) or directly measured (32), and the results presented in Supplementary Material as indicated. To convert LDL-C from mg/dL to mmol/L, the value was multiplied by a conversion factor of 0.02586. Non-HDL-C was calculated by subtracting HDL-C from TC. HbA1c was measured using NGSP certified, IFCC traceable assays (Boronate affinity chromatography and turbidimetric inhibition immunoassay).

### 2.5 Statistical analysis

Descriptive statistics are presented as medians with interquartile ranges (IQR) for continuous variables, and frequencies and proportions (%) for categorial variables unless otherwise indicated. Chi-Square test was used to assess differences in frequencies. Fisher’s exact test was used in pair-wise comparisons when the cell count of one or more cells of the contingency table was less than five. Differences in continuous variables across ASCVD risk categories was assessed using ANOVA (variables that failed the Kolmogorov-Smirnov test were log-transformed prior to analysis) with Welch’s t-test in cases of significant variance heterogeneity with Games-Howell test for pair-wise *post-hoc* analysis. In exploratory analyses, univariate logistic regression models was used to determine if sex (female vs male) and age (> vs ≤ median age) was associated with LLT (yes/no). Analysis was additionally adjusted for age (years)/sex as appropriate, diabetes duration (years), HbA1c (%), and ASCVD risk (moderate, high, or very high). A *P*-value <0.05 was considered statistically significant, and no adjustment for multiple comparisons were done. Analysis was done using SAS version 9.4.

## 3 Results

Of 322 eligible patients, 106 (32.9%) were excluded due to missing data (the majority missing apoB). Characteristics of the 216 patients included in the analysis are shown in **Table 2**. The median (IQR) age was 43.6 (31.9-58.2) years, diabetes duration 16 (10-25) years, HbA1c 7.3 (6.8-8.0)% (56 [51–64] mmol/mol), and 39.4% were females.

**Table 2:** Patient characteristics, further stratified by atherosclerotic cardiovascular disease risk.

		ASCVD risk		
Characteristic	All (n=216)	Moderate (n=29)	High (n=162)	Very high (n=25)
Females	39.4	34.5	42.6	24.0
Age, yrs	43.6 (31.9-58.2)	39.8 (27.6-49.0)	42.8 (31.4-55.7)	62.3 (54.8-72.8)
Years since diagnosis, yrs	16 (10-25)	7 (4-8)	18 (12-25)	22 (14-41)
HbA1c, %	7.3 (6.8-8.0)	7.1 (6.6-7.8)	7.3 (6.8-8.0)	7.2 (7.0-8.2)
HbA1c, mmol/mol	56 (51-64)	54 (49-62)	56 (51-64)	55 (53-66)
BMI, kg/m^2^	25.2 (23.0-27.9)	24.2 (22.7-26.0)	25.5 (23.4-28.2)	25.2 (22.2-27.3)
Systolic BP, mmHg	128 (118-138)^a^	124 (115-131)	128 (120-138)	131 (119-147)^a^
Diastolic BP, mmHg	78 (73-84)^a^	77 (72-81)	79 (74-84)	74 (68-83)^a^
Hypertension	27.8	–	24.7	80.0
Current smoker	19.0	–	21.0	28.0
Lipid levels, mmol/L				
Triglycerides	0.9 (0.7-1.1)	0.8 (0.6-1.0)	0.9 (0.7-1.1)	1.0 (0.8-1.4)
Total cholesterol	4.8 (4.1-5.5)	5.0 (4.4-5.6)	4.8 (4.1-5.5)	3.7 (3.2-4.9)
HDL-cholesterol	1.5 (1.3-1.8)	1.6 (1.4-1.9)	1.6 (1.3-1.8)	1.4 (1.2-1.7)
LDL-cholesterol, measured	2.8 (2.3-3.4)	3.1 (2.6-3.4)	2.8 (2.4-3.4)	2.1 (1.8-2.6)
LDL-cholesterol, Friedewald	2.7 (2.1-3.3)	3.1 (2.6-3.3)	2.7 (2.2-3.4)	1.9 (1.5-2.6)
LDL-cholesterol, Sampson	2.7 (2.2-3.4)	3.1 (2.6-3.4)	2.7 (2.2-3.5)	1.9 (1.5-2.6)
Non-HDL cholesterol	3.1 (2.5-3.8)	3.4 (3.0-3.8)	3.1 (2.6-3.9)	2.3 (2.1-3.0)
ApoB, g/L	0.75 (0.60-0.90)	0.81 (0.69-0.87)	0.77 (0.60-0.92)	0.60 (0.55-0.68)
Diabetes-related complications				
Nephropathy	16.7	–	13.0	60.0
Neuropathy	14.8	–	11.7	52.0
Retinopathy	18.1	–	16.7	48.0
CVD	7.4	–	–	64.0
Myocardial infarction	2.8	–	–	24.0
Stroke	0.5	–	–	4.0
Lipid-lowering therapy	33.6^b^	17.2	29.4^b^	80.0
Statin	32.2^b^	17.2	29.4^b^	76.0
Low intensity	1.4	–	2.1	–
Medium intensity	40.9	40.0	44.7	31.6
High intensity	57.8	60.0	53.2	68.4
Ezetimibe	14.0^b^	6.9	10.6^b^	44.0
PCSK9 inhibitor	0.5^b^	–	–	4.0
Bempedoic acid	0.5^b^	–	–	4.0
Antihypertensive therapy	26.2^b^	–	22.5^b^	80.0

Data are presented as median (IQR) or percentage. ApoB, apolipoprotein B; ASCVD, atherosclerotic cardiovascular disease; BMI, body mass index; BP, blood pressure; CVD, cardiovascular disease; HbA1c, glycated hemoglobin; LDL, low-density lipoprotein; HDL, high-density lipoprotein; PCSK9, proprotein convertase subtilisin/kexin type 9. ^a^ information missing in one patient ^b^ information missing in two patients.

Based on ESC 2021, 13.4% of the patients were at moderate, 75.0% at high, and 11.6% at very high ASCVD risk. LLT was prescribed to 33.6% (n=72) of the patients, of which 98.6% (n=71) received statin and 41.7% (n=30) ezetimibe. Information on medication was missing for two patients. A linear trend in the prescription of LLT was observed across ASCVD risk; patients at very high risk were more likely to be prescribed treatment than patients at high or moderate risk (80.0% vs 29.4% and 17.2%, respectively, both *P*-values <0.00001). Whereas no significant differences in statin intensity was observed, patients at very high risk where more likely to be treated with ezetimibe (44.0% vs 10.6% and 6.9%, respectively, both *P*-values <0.005) and was the only group where PCSK9i were prescribed (one patient). In line with this, TC, LDL-C, HDL-C, non-HDL-C, and apoB levels tended to be lowest among patients at very high risk, and highest among patients at moderate risk (**Table 2**). The only differences that reached statistical significance was lower LDL-C (regardless of method of assessment) and non-HDL-C levels among patients at very high as compared to high and moderate risk, as well as lower TC and higher TG levels compared to patients at moderate risk (all *P*-values <0.05).

### 3.1 Lipid target attainment

**Figure 1:**
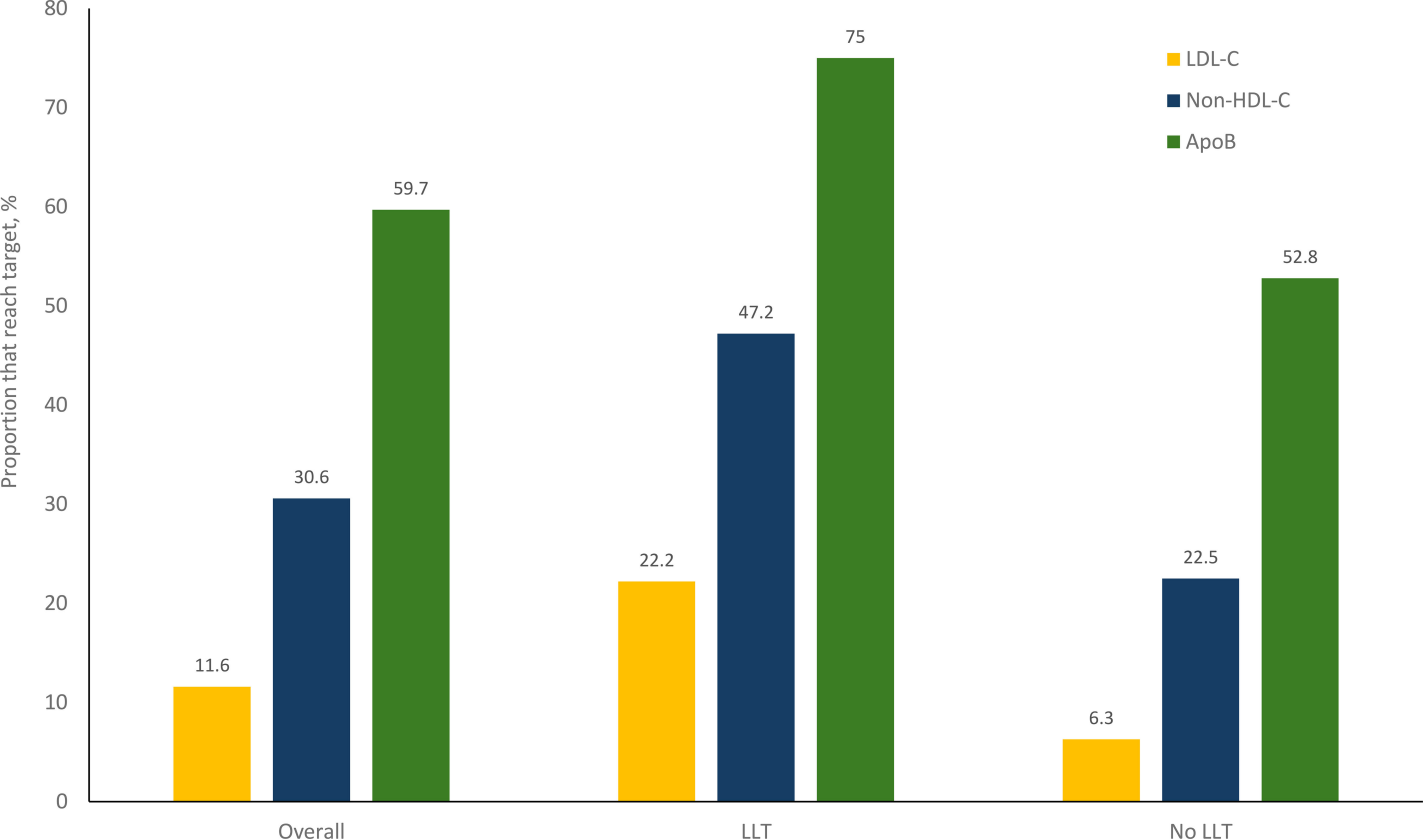
Proportion of the 216 patients that reached the LDL-cholesterol, non-HDL-cholesterol, and apoB targets recommended by the European Society of Cardiology in the 2021 guideline on cardiovascular disease prevention in clinical practice (21), further stratified by lipid-lowering therapy (LLT) (n=72) and no LLT (n=142). More detailed information on the target recommendations is available in **Table 1**. Information on medication missing in two patients.

**Figure 2:**
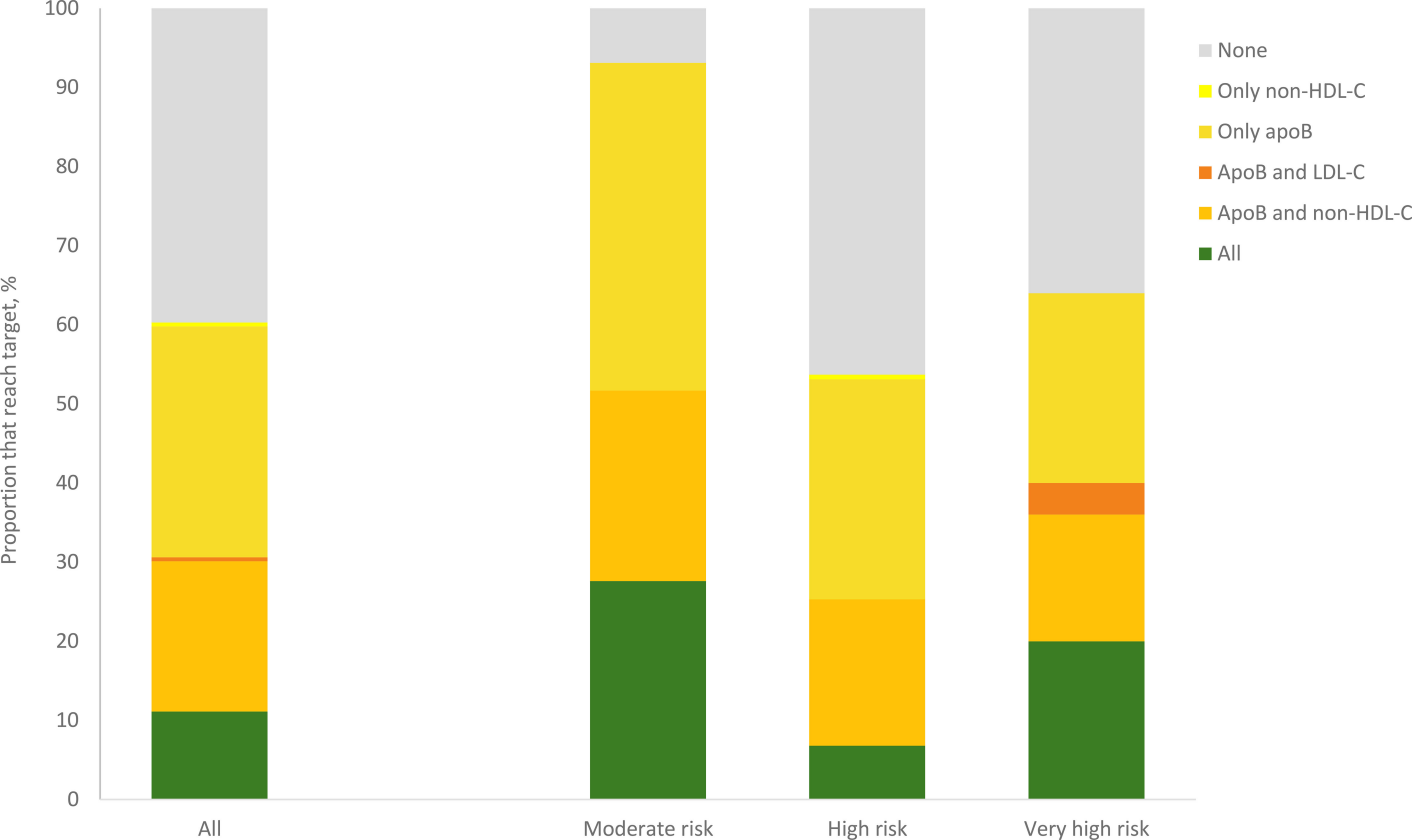
Agreement in target attainment across LDL-cholesterol, non-HDL-cholesterol, and apoB overall among 216 patients with type 1 diabetes mellitus, further stratified by atherosclerotic cardiovascular disease risk (moderate, high, and very high). More detailed information on the specific targets recommended by the European Society of Cardiology is available in **Table 1**, and the definitions of atherosclerotic cardiovascular disease risk are available in **Supplementary Table 1**.

The recommended LDL-C target was met by 11.6% of the patients. Significantly more patients reached the non-HDL-C and apoB target (30.6% and 59.7%, respectively), where 2.6 times more patients reached the non-HDL-C target, and 5.1 times more patients reached the apoB target compared to LDL-C (all *P*-values <0.00001) (**Figure 1**) (**Supplementary Table 2**). Agreement across lipid biomarkers overall and further stratified by ASCVD risk are shown in **Figure 2**. Overall, 11.1% of the patients reached all three, and 39.8% none of the lipid targets (**Supplementary Tables 3–6**).

Only marginal changes to the results were observed when considering directly measured LDL-C or LDL-C estimated by the Sampson formula (**Supplementary Tables 2–6**). Overall, LDL-C target attainment based on the Friedewald and Sampson formula yielded very similar results (11.6% vs 10.7%) and was slightly lower based on directly measured LDL-C (7.9%), with no statistically significant differences observed across the three assessment methods (*P*-value=0.43).

### 3.2 Secondary analysis

#### 3.2.1 Lipid lowering medication

Patients prescribed LLT were more likely to reach the recommended lipid targets compared to patients without LLT (LDL-C, 22.2% vs 6.3%; non-HDL-C, 47.2% vs 22.5%; apoB, 75.0% vs 52.8%, all *P*-values ≤0.001) (**Figure 1**, **Supplementary Table 7**). In terms of agreement in target attainment across lipid biomarkers, significantly fewer patients on LLT than without LLT failed to reach any of the targets (25.0% vs 46.5%, *P*-value=0.002) and significantly more patients achieved all three lipid targets, (20.8% vs. 6.3%, *P* = 0.001) (**Supplementary Table 8**). Similar results were obtained with Sampson and directly measured LDL-C levels, although the difference in the proportion of patients that reached all three lipid targets was no longer statistically significant with directly measured LDL-C (**Supplementary Tables 9, 10**).

#### 3.2.2 Sex differences

ASCVD risk tended to be differently distributed among males and females, with more male patients at very high risk (14.5% vs 7.1%, *P*-value=0.09) and more female patients at high risk (81.2% vs 71.0%, *P*-value=0.09), with a similar proportion at moderate risk (11.8% females and 14.5% males, *P*-value=0.56). Although no statistically significant differences in lipid target attainment was observed between sexes (**Supplementary Table 11**), females were significantly less likely to be prescribed LLT than males (21.4% vs 41.5%, *P*-value=0.002) (**Supplementary Tables 12**). In univariate analysis, the odds of LLT for female patients was 0.38 times that of male patients (OR = 0.38, 95% CI [0.21, 0.72]), which remained statistically significant after adjusting for age, diabetes duration, HbA1c, and ASCVD risk (OR = 0.37, 95% CI [0.17, 0.83]) (**Supplementary Table 13**).

#### 3.2.3 Age differences

Patients above median age (43.6 years) were more likely to be prescribed LLT than patient at or below median age (55.6% vs 11.3%, *P*-value <0.00001) (**Supplementary Table 14**). Although this is in line with more patients above median age being at very high risk (19.4% vs 3.7%, *P*-value=0.0004) and less patients being at moderate risk (8.3% vs 18.5%, *P*-value=0.03), this was also observed within ASCVD risk categories (high risk, 47.4% >43.6 years vs 12.2% ≤43.6 years, and very high risk, 95.2% >43.6 years vs 0% ≤43.6 years, both *P*-values <0.001) (**Supplementary Tables 14, 15**). In univariate analysis, the odds of LLT for patients above median age was 10.0 times higher than for patients at or below median age (OR = 10.00, 95% CI [4.92, 20.34]), which remained statistically significant after adjusting for sex, diabetes duration, HbA1c, and ASCVD risk (OR = 7.50, 95% CI [3.53, 15.96]) (**Supplementary Table 13**). Nevertheless, no significant differences in lipid target attainment were observed (**Supplementary Table 15**).

#### 3.2.4 Included versus excluded patients

Comparing basic clinical characteristics of included (n=216) and excluded (n=106) patients, no differences in age, systolic or diastolic blood pressure, TG and HDL-C levels, or proportion of females or active smokers were observed. However, excluded patients had slightly longer diabetes duration (19 [12–28] years vs 16 [10–25] years, *P*-value=0.03), better controlled HbA1c (7.0 [6.4-7.8]% vs 7.3 [6.8-8.0]% (53 [46–62] mmol/mol vs. 56 [51–64] mmol/mol), *P*-value=0.02), and lower LDL-C (2.2 [1.6-2.8] mmol/L vs 2.7 [2.1-3.3] mmol/L, *P*-value <0.0001) and non-HDL-C (2.6 [2.0-3.3] mmol/L vs 3.1 [2.5-3.8] mmol/L, *P*-value <0.0001) (**Supplementary Table 16**). ASCVD risk could be determined for 94.3% (n=100) of the excluded patients (5% at moderate, 84% at high, and 11% at very high risk), and the distribution did not significantly differ from the included patients (*P*-value>0.05). A similar proportion of included and excluded patients was prescribed LLT (33.6% and 39.8%, *P*-value>0.05, information on medication was missing for eight excluded patients).

Of the excluded patients for which LDL-C (n=93) and non-HDL-C (n=90) target attainment could be determined, 24.7% and 52.2% reached the recommended target, respectively, which was significantly higher than the 11.6% and 30.6% observed in the main analysis (both *P*-values <0.05). A similar discordance in target attainment was observed among the 90 patients for which both LDL-C and non-HDL-C target attainment could be determined (25.6% vs 52.2%, *P*-value=0.0002). Compared to the 216 patients included in the analysis, fewer of the excluded patients failed to reach any of the targets (46.7% vs 69.0% (*P*-value=0.0002), more patients reached both targets (24.4% vs 11.1% (*P*-value=0.003), whereas no differences were observed in the proportion of patients that only reached one target (only LDL-C, 1.1% vs 0.5%, *P*-value=0.52; only non-HDL-C, 27.8% vs 19.4%, *P*-value=0.11).

## 4 Discussion

The results underscore that most adult patients with T1DM (>86%) are classified as having high or very high ASCVD risk according to the current ESC framework. Despite this, only one in ten patients met the recommended LDL-C target, and half of the patients presented with LDL-C ≥2.7 mmol/L. This is largely explained by the small proportion (34%) of patients prescribed LLT. If non-HDL-C and apoB were considered in addition to LDL-C, target attainment varied notably across the three lipid biomarkers. While LDL-C target attainment was markedly low, significantly more patients met the recommended targets for non-HDL-C and apoB, regardless of LLT.

Although limited data is currently available, the finding that patients presented with a lipid phenotype characterized by elevated LDL-C levels but more normal non-HDL-C and apoB levels is in line with previous observations in T1DM, although no study has to our knowledge compared target attainment across all three lipid biomarkers. In a recent study based on registry data from 2020-2021, Brandts et al. compared LDL-C and non-HDL-C target attainment in accordance with the ESC 2019/2021 recommendations (20, 21) among 8314 patients with T1DM. A discrepancy in target attainment was observed at all levels of ASCVD risk; at moderate risk, 67.9% and 89.3% of the patients reached recommended LDL-C and non-HDL-C targets, respectively, at high risk 8.8% and 22.7%, and at very high risk 4.5% and 14.3% (27). Whereas the results among patients at high ASCVD risk were similar to that observed in SwissDiab, absolute target attainment was higher among patients at moderate risk, which were considerably younger than the SwissDiab patients (20.6 [18.5-20.7] years vs 39.8 [27.6-49.0] years), but with similar diabetes duration (5 [3–8] years vs 7 [4–8] years). Target attainment was lower among patients at very high risk, which is likely the result of fewer patients being prescribed LLT (32% vs 80%). In a study from 2009, based on 169 consecutive and consenting adult outpatients with T1DM without LLT, 66% of female, and 62% of male patients that failed to reach the LDL-C target (≤2.0 mmol/L) recommended by the Canadian Diabetes Association reached the apoB target (<0.90 g/L) (28). A study in 2015 among 652 adult patients with T1DM included in the Coronary Artery Calcification in Type 1 Diabetes Study (CACTI) showed that 31.6% had elevated non-HDL-C (≥130 mg/dl, ~3.4 mmol/L) and 47.9% elevated apoB levels (≥0.9 g/L). Whereas 50.6% of the patients fulfilled both the non-HDL-C and apoB target, 17.8% reached only the non-HDL-C, and 1.5% only the apoB target (29).

These findings are in contrast to T2DM, where elevated non-HDL-C and apoB levels despite normal LDL-C levels are common (33). This discrepancy highlights important pathophysiological differences between T1DM and T2DM. Previous literature has supported the use of non-HDL-C and apoB as alternative or even superior biomarkers to LDL-C for cardiovascular risk assessment, particularly in patients with atherogenic dyslipidemia (12, 33–35). The 2019 ESC/EASD and ESC 2021 guidelines recommend the use of non-HDL-C and apoB as secondary treatment targets, especially in patients with hypertriglyceridemia, a common feature in T2DM (19, 21). It remains unclear to what extent these recommendations apply to patients with T1DM and raises important questions regarding the appropriateness of relying solely on LDL-C to guide CVD prevention in this patient population, as it might lead to overtreatment in patients with otherwise favorable apoB and/or non-HDL-C levels. The CACTI study showed that patients with T1DM and elevated non-HDL-C and apoB levels had significantly greater odds of progression of coronary artery calcification over six years than patients with normal non-HDL-C and apoB levels (OR = 1.90, 95% CI [1.15-3.15]), or only elevated apoB (OR = 2.86, 95% CI [1.43-5.74]) (29). These results suggest that apoB and non-HDL-C are complementary rather than mutually exclusive cardiovascular risk markers. Although available data is limited and often based on observational studies, the results illustrate the importance of a better understanding of the interrelationship between LDL-C, non-HDL-C, apoB, and cardiovascular risk in T1DM.

Other studies caution against focusing solely on the quantitative aspects of lipoproteins in T1DM. This, as patients with T1DM tend to exhibit significant qualitative and functional abnormalities with respect to lipoprotein particles, which contribute to the increased atherogenic risk observed in this patient population (1, 36). This masked risk, often not captured by standard lipid panels, might lead to underestimation of cardiovascular risk (35). This underscores the need for further research to increase our understanding of the clinical utility of different lipid biomarkers in T1DM.

Several factors might have contributed to the low LDL-C target attainment. One reason is underutilization of LLT, which is commonly reported in this patient population (37–40). A contributing factor might be clinical inertia affecting younger patients (half of the patients were below 43 years of age) for whom ASCVD risk may be underestimated, particularly in the context of good glycemic control (38). That patients above median age were more likely to be prescribed LLT is in line with a greater proportion being at very high cardiovascular risk. However, even within each cardiovascular risk category, patients above 43 years of age were more likely to be prescribed LLT, indicating that age might be influencing treatment decisions. A similar finding was observed in a retrospective clinical audit at the Diabetes and Endocrinology Department at Hull Royal Infirmary. Of the adult patients with T1DM that met the NICE criteria for primary CVD prevention, 43% of patients <40 years of age were prescribed statin, compared to 73% of patients ≥40 years (41). Similar results were found in a recent study examining age-specific inequalities in statin initiation among adults with elevated ASCVD risk (42). In addition, patient-related factors including statin-related side effects and limited self-awareness of cardiovascular risk, and thus the benefit of treatment, may further hinder optimal lipid management (37, 43). Furthermore, the historical underrepresentation of individuals with T1DM in major cardiovascular prevention trials and treatment guidelines has likely contributed to an uncertainty among treating physicians regarding the optimal lipid management strategy in this patient population (38, 44).

Although women with T1DM carry a twofold increased cardiovascular risk compared to men (45), females were less likely to be prescribed LLT. While the majority of research has focused on T2DM, consistent sex-based inequalities in lipid management have been reported in both T1DM and T2DM (46–50). In a retrospective study based on data on 266 adult patients with T1DM in quaternary care across northeastern Ohio in 2020-2024, the odds of female patients receiving statin therapy was 0.45, 95% CI [0.24, 0.85] (adjusted for age, race and history of ASCVD (40), as compared to 0.37, 95% CI [0.17, 0.83] observed in the current study. A similar tendency was observed in data from 2020-2022, among younger patients with longer diabetes duration without prior history of ASCVD. Of the 2,657 patients (37 [28–52] years and 21 [12–32] years diabetes duration) from the multicenter Prospective Diabetes Follow-up Registry (DPV) in Germany, females with LDL-C ≥3.4 mmol/L were less likely than males with LDL-C ≥3.4 mmol/L to be prescribed statin (7.9% vs 17.0%, P<0.01). This was not observed among the 1,172 patients (36 [27–49] years and 21 [12–30] years diabetes duration) from the Société Francophone du Diabète– Cohorte Diabète de Type 1 (SFDT1), where 18.2% of females, and 21.0% of males with LDL-C ≥3.4 mmol/L were prescribed statin, p=0.78) (51). Results from a population-based retrospective study based on primary healthcare data in Australia confirm the overall poor LDL-C control in T1DM. Among 910 patients, 8.6% of females (mean [SD] age 46.9 [18.3] years) and 7.5% of males (47.3 [16.9] years) reached an LDL-C target of <2.0 mmol/L or <1.8 mmol/L in the presence of ASCVD, with no statistically significant difference in LLT observed among females and males (23.3% vs 28.6%) (50). Reluctance to prescribe statin to women of child-bearing age due to fear of birth defects and a higher risk of miscarriage may play a role, although causality has never been established, and despite decades of experience showing absence of harm in cases where statin was unwittingly used before and during pregnancy (52). Another contributing factor may be an increased prevalence of statin-related muscle symptoms and intolerance in women, who have been shown to be more likely to stop or switch statin treatment (53, 54). Also of importance is likely the common notion that women have lower cardiovascular risk than men, which partly goes back to sex-specific difference in risk factors, hormonal protection, and a more favorable lipid profile (55). Although CVD tends to develop 7–10 years later in women than men (56), the presence of diabetes has been shown to significantly increase the risk of CVD approximately 2–7 fold in women and 2–3 fold in men, even under conditions of good glycemic control (45, 57). Women with T1DM bear a disproportionately higher burden of CVD and experience a greater loss of life-years (approximately 18 years versus 14 years in men) (58, 59). A higher risk of non-attainment of LDL-C targets among women with diabetes has been shown in several studies (46, 48, 50, 54), which was not observed in the current study. This might be attributable to our relatively small sample size and the low LDL-C target attainment overall, the increased stringency of recommended LDL-C targets which has affected target attainment over time, and the limited efficacy of statin monotherapy in achieving currently recommended LDL-C targets.

The Friedewald formula has been shown to underestimate cardiovascular risk compared to newer methods, like the Sampson formula (60). In general, the methods used to determine LDL-C levels in this study (determined with the Friedewald and Sampson formula and directly measured) did not meaningfully influence target attainment and thus clinical decision-making.

The strength of the current study lies in the real-world clinical setting, with SwissDiab being integrated into routine clinical practice. Although the generalizability of the results to other tertiary care settings is likely considerable, patient selection bias should always be considered as patients that participate in clinical studies tend to have a better overall health status and be more engaged in their care. This was also observed in a previous SwissDiab study. Comparing characteristics of patients enrolled in SwissDiab with non-participating patients, the former had a slightly better controlled diabetes. Although a better lipid profile was observed among SwissDiab participants in T2DM, this was not the case in T1DM (61). The current results are thus more likely to slightly overestimate than underestimate lipid management in the overall patient population. Given that SwissDiab is set within tertiary care centers in Switzerland with a particularly strong research interest, they might not provide an accurate representation of diabetes care in general. Smaller hospitals, as well as secondary and primary care settings, were not included. However, given the complexity of treatment, the majority of patients with T1DM are treated by an endocrinologist/diabetologist in a private or tertiary care setting. Consequently, the findings may not be generalizable to all tertiary care settings, or T1DM care in general in Switzerland, and might overestimate lipid management outcomes relative to the overall patient population. In addition, SwissDiab only includes patients ≥18 years, limiting the generalizability of the findings to younger patient populations.

The relatively small sample size, particularly in stratified analysis, has limited statistical power, and the reported differences in LLT based on sex and age are to be considered exploratory. Although the excluded patients had similar age, proportion of females, distribution of ASCVD risk, and prescription of LLT, they presented with better glycemic control and significantly lower non-HDL-C and LDL-C levels (9.4% missing data). In line with this, excluded patients were more likely to reach recommended LDL-C and non-HDL-C targets than included patients, although the proportion that reached LDL-C target remained low (less than one in three patients). While this indicates that the current analysis is affected by selection bias and underestimates overall lipid target attainment in this patient population, it remains inadequate. Although apoB was not available in the excluded patients, the observed inconsistency in target attainment between LDL-C and non-HDL-C support the finding of poor consistency in target attainment across lipid biomarkers. Furthermore, treatment naïve LDL-C was extrapolated based on current LLT, an approach commonly used in research studies and adopted in clinical guidelines (20, 62). Although inter-individual variation in treatment response to drugs are well recognized, the assumptions made are based on reported averages, and any over- and underestimations of LLT-associated reductions in LDL-C are likely to even out. This is supported by a previous study, showing that the median (IQR) difference between baseline LDL-C retrieved from medical records and estimated based on the current LLT among 137 patients with T2DM was 0 (-0.8, 0.9) mmol/L (63). A similar proportion of under- and over-estimation of the relative LDL-C target can thus be assumed. In the current analysis, only four patients (all at high ASCVD risk) fulfilled the absolute LDL-C target (<1.8 mmol/L) but not the ≥50% reduction target. Three of these patients were prescribed medium intensity statin, and one patient low intensity statin + ezetimibe, treatments that are unlikely to reduce LDL-C levels by 50% or more. Taken together, the method used to estimate treatment naïve LDL-C is unlikely to have significantly influenced the proportion of patients considered at target in the current analysis. Although the results show that some patients display a marked inconsistency in target attainment across LDL-C, non-HDL-C, and apoB, the cross-sectional design does not allow for assessment of long-term cardiovascular outcomes associated with different lipid target attainment patterns. Finally, in the absence of clear guidelines on lipid management in T1DM, the cardiovascular risk definitions and lipid targets applied were primarily established for T2DM, which might not fully capture the nuances of cardiovascular risk and optimal lipid management in T1DM. Given the criteria used, this is more likely to have underestimated than overestimated cardiovascular risk. These limitations do not mitigate the overall conclusion that there is a need for improved awareness of cardiovascular risk and lipid management in patients with T1DM, although the appropriate lipid targets in this patient population might need further exploration, as suggested by the current results.

In conclusion, the current findings confirm the high ASCVD risk and overall need to raise awareness of lipid management as one key cornerstone in T1DM care. Markedly inconsistent agreement in LDL-C, non-HDL-C, and apoB target attainment was observed, which can create uncertainties in clinical decision-making on how to best apply current lipid management recommendations for optimal cardiovascular risk reduction in patients with T1DM.

## Data Availability

The datasets presented in this article are not readily available, as this is not approved by the participants within the framework of the informed consent, and because of possible identification of patients by individuals or organizations with access to overlapping datasets. Requests to access the datasets should be directed to Michael.braendle@h-och.ch.
