# Supplementary material for: Patients with type 1 diabetes mellitus and recommended low-density lipoprotein cholesterol, non-high-density lipoprotein cholesterol and apolipoprotein B targets – a SwissDiab study
Source: Front Endocrinol (Lausanne). 2026 May 29;17:1819019. doi: 10.3389/fendo.2026.1819019 (PMC13259660; doi:10.3389/fendo.2026.1819019)
Supplement: Supplementary file 1 [file DataSheet1.docx]

**Supplemental Tables and Figures**

***Table of content***

[**Suppl. Table 1.** Definition of ASCVD risk in patients with T1DM based on the ESC. 2](#_Toc215605013)

[**Suppl. Table 2.** The proportion of patients that reach the ESC lipid targets overall and stratified by ASCVD risk. 2](#_Toc215605014)

[**Suppl. Table 3.** Agreement in target attainment across recommended lipid biomarkers. 3](#_Toc215605015)

[**Suppl. Table 4.** Agreement in target attainment across recommended lipid biomarkers in patients at moderate ASCVD risk. 3](#_Toc215605020)

[**Suppl. Table 5.** Agreement in target attainment across recommended lipid biomarkers in patients at high ASCVD risk. 4](#_Toc215605021)

[**Suppl. Table 6.** Agreement in target attainment across recommended lipid biomarkers in patients at very high ASCVD risk. 4](#_Toc215605022)

[**Suppl. Table 7.** The proportion of patients that reach lipid targets, overall and further stratified by lipid-lowering therapy. 5](#_Toc215605016)

[**Suppl. Table 8.** Agreement in target attainment across recommended lipid biomarkers stratified by lipid-lowering therapy – LDL-cholesterol estimated based on Friedewald. 5](#_Toc215605017)

[**Suppl. Table 9.** Agreement in target attainment across recommended lipid biomarkers stratified by lipid-lowering therapy – LDL-cholesterol estimated based on Sampson. 6](#_Toc215605018)

[**Suppl. Table 10.** Agreement in target attainment across recommended lipid biomarkers stratified by lipid-lowering therapy – directly measured LDL-cholesterol. 6](#_Toc215605019)

[**Suppl. Table 11.** The proportion of patients that reach the ESC lipid targets overall and stratified by sex. 7](#_Toc215605023)

[**Suppl. Table 12.** The proportion of patients on lipid-lowering therapy stratified by ASCVD risk and sex. 7](#_Toc215605024)

[**Suppl. Table 13.** Associations between age and sex and prescription of lipid-lowering medication. 8](#_Toc215605025)

[**Suppl. Table 14.** The proportion of patients on lipid-lowering therapy stratified by ASCVD risk and median age. 9](#_Toc215605025)

[**Suppl. Table 15.** The distribution of ASCVD risk and the proportion of patients that reach ESC lipid targets stratified by median age. 9](#_Toc215605026)

[**Suppl. Table 16.** Basic characteristics of included and excluded patients 10](#_Toc215605027)

**Suppl. Table 1.** Definition of ASCVD risk in patients with T1DM based on the ESC.

| Moderate Risk | - diabetes duration <10 years, without target organ damage or other major risk factors |
| --- | --- |
| High risk | - Patients that are not identified as having moderate or very high risk |
| Very high risk | - Established ASCVD - Severe target organ damage   - eGFR <45 ml/min/1.73 m^2^  - eGFR 45-59 ml/min/1.73 m^2^ and microalbuminuria (3 < ACR ≤30 mg/mmol)  - macroalbuminuria (ACR >30 mg/mmol)  - microvascular disease in at least 3 different sites (e.g. retinopathy + microalbuminuria + neuropathy)   - LDL-cholesterol >5 mmol/L |
| Major risk factors | - Hypertension - Smoking - Dyslipidemia |
| Established  ASCVD | - Myocardial infarction - Coronary revascularization (PTCA, CABG) - Stroke - Peripheral artery disease |

The current definitions are a slightly modified version of the definitions provided in the 2021 guideline by the European Society of Cardiology (Visseren et al, Eur Heart J, 2021). ACR, albumin-creatinine ratio; ASCVD, atherosclerotic cardiovascular disease; CABG, coronary artery bypass grafting; eGFR, estimated glomerular filtration rate; ESC, European Society of Cardiology; LDL, low-density lipoprotein; PTCA, percutaneous transluminal coronary angioplasty; T1DM, type 1 diabetes mellitus.

**Suppl. Table 2.** The proportion of patients that reach the ESC lipid targets overall and stratified by ASCVD risk.

|  | All  (n=216) | |  | Moderate (n=29) | |  | High  (n=162) | |  | Very high (n=25) | | *P*-diff** |
| --- | --- | --- | --- | --- | --- | --- | --- | --- | --- | --- | --- | --- |
| Target attainment | n | % |  | n | % |  | n | % |  | n | % |  |
| LDL-C, Friedewald^*^ | 25 | 11.6 |  | 8 | 27.6 |  | 11 | 6.8 |  | 6 | 24.0 | 0.0007 |
| LDL-C, Sampson^*^ | 23 | 10.7 |  | 8 | 27.6 |  | 10 | 6.2 |  | 5 | 20.0 | 0.0007 |
| LDL-C, measured^*^ | 17 | 7.9 |  | 8 | 27.6 |  | 7 | 4.3 |  | 2 | 8.0 | 0.0001 |
| Non-HDL-C cholesterol | 66 | 30.6 |  | 15 | 51.7 |  | 42 | 25.9 |  | 9 | 36.0 | 0.017 |
| ApoB | 129 | 59.7 |  | 27 | 93.1 |  | 86 | 53.1 |  | 16 | 64.0 | 0.0003 |

ApoB, apolipoprotein B; C, cholesterol; ESC, European Society of Cardiology. ^*^ Baseline LDL-cholesterol extrapolated based on average effect of current lipid-lowering therapy.

^**^ Difference between ESC ASCVD risk categories determined by Chi-Square test.

**Suppl. Table 3.** Agreement in target attainment across recommended lipid biomarkers.

|  | LDL-cholesterol | | |
| --- | --- | --- | --- |
| Target attainment | Friedewald | Sampson | Measured |
| None | 86 (39.8) | 86 (39.8) | 86 (39.8) |
| All three | 24 (11.1) | 23 (10.7) | 17 (7.9) |
| Only LDL-C | - | - | - |
| Only non-HDL-C | 1 (0.5) | 1 (0.5) | 1 (0.5) |
| Only ApoB | 63 (29.2) | 64 (29.6) | 64 (29.6) |
| LDL-C and Non-HDL-C | - | - | - |
| LDL-C and ApoB | 1 (0.5) |  | - |
| Non-HDL-C and ApoB | 41 (19.0) | 42 (19.4) | 48 (22.2) |

Data provided are frequency (percentage), n=216. ApoB, apolipoprotein B;

C, cholesterol; HDL, high-density lipoprotein; LDL, low-density lipoprotein.

**Suppl. Table 4.** Agreement in target attainment across recommended lipid biomarkers in patients at moderate ASCVD risk.

|  | LDL-cholesterol | | |  |
| --- | --- | --- | --- | --- |
| Target attainment | Friedewald | Sampson | Measured | |
| None | 2 (6.9) | 2 (6.9) | 2 (6.9) | |
| All three | 8 (27.6) | 8 (27.6) | 8 (27.6) | |
| Only LDL-C | - | - | - | |
| Only non-HDL-C | - | - | - | |
| Only ApoB | 12 (41.4) | 12 (41.4) | 12 (41.4) | |
| LDL-C and non-HDL-C | - | - | - | |
| LDL-C and ApoB | - | - | - | |
| Non-HDL-C and ApoB | 7 (24.1) | 7 (24.1) | 7 (24.1) | |

Data provided are frequency (percentage). ApoB, apolipoprotein B; ASCVD, atherosclerotic cardiovascular disease; C, cholesterol; HDL, high-density lipoprotein; LLT, lipid-lowering therapy; LDL, low-density lipoprotein. See Supplemental Table 1 for definition of moderate ASCVD risk.

**Suppl. Table 5.** Agreement in target attainment across recommended lipid biomarkers in patients at high ASCVD risk.

|  | LDL-cholesterol | | |  |
| --- | --- | --- | --- | --- |
| Target attainment | Friedewald | Sampson | Measured | |
| None | 75 (46.3) | 75 (46.3) | 75 (46.3) | |
| All three | 11 (6.8) | 10 (6.2) | 7 (4.3) | |
| Only LDL-C | - | - | - | |
| Only non-HDL-C | 1 (0.6) | 1 (0.6) | 1 (0.6) | |
| Only ApoB | 45 (27.8) | 45 (27.8) | 45 (27.8) | |
| LDL-C and non-HDL-C | - | - | - | |
| LDL-C and ApoB | - | - | - | |
| Non-HDL-C and ApoB | 30 (18.5) | 31 (19.1) | 34 (21.0) | |

Data provided are frequency (percentage). ApoB, apolipoprotein B; ASCVD, atherosclerotic cardiovascular disease; C, cholesterol; HDL, high-density lipoprotein; LLT, lipid-lowering therapy; LDL, low-density lipoprotein. See Supplemental Table 1 for definition of moderate ASCVD risk.

**Suppl. Table 6**. Agreement in target attainment across recommended lipid biomarkers in patients at very high ASCVD risk.

|  | LDL-cholesterol | | |
| --- | --- | --- | --- |
| Target attainment | Friedewald | Sampson | Measured |
| None | 9 (36.0) | 9 (36.0) | 9 (36.0) |
| All three | 5 (20.0) | 5 (20.0) | 2 (8.0) |
| Only LDL-C | - | - | - |
| Only non-HDL-C | - | - | - |
| Only ApoB | 6 (24.0) | 7 (28.0) | 7 (28.0) |
| LDL-C and non-HDL-C | - | - | - |
| LDL-C and ApoB | 1 (4.0) | - | - |
| Non-HDL-C and ApoB | 4 (16.0) | 4 (16.0) | 7 (28.0) |

Data provided are frequency (percentage). ApoB, apolipoprotein B; ASCVD, atherosclerotic cardiovascular disease; C, cholesterol; HDL, high-density lipoprotein; LLT, lipid-lowering therapy; LDL, low-density lipoprotein. See Supplemental Table 1 for definition of moderate ASCVD risk.

**Suppl. Table 7.** The proportion of patients that reach lipid targets, overall and further stratified by lipid-lowering therapy.

|  | All  (n=214) | |  | LLT  (n=72) | |  | No LLT  (n=142) | |  |  |
| --- | --- | --- | --- | --- | --- | --- | --- | --- | --- | --- |
| Target attainment | n | % |  | n | % |  | n | % |  | *P*-diff^*^ |
| LDL-C, Friedewald | 25 | 11.6 |  | 16 | 22.2 |  | 9 | 6.3 |  | 0.0005 |
| LDL-C, Sampson | 23 | 10.7 |  | 14 | 19.4 |  | 9 | 6.3 |  | 0.003 |
| LDL-C, measured | 17 | 7.9 |  | 9 | 12.5 |  | 8 | 5.6 |  | 0.08 |
| Non-HDL-C | 66 | 30.6 |  | 34 | 47.2 |  | 32 | 22.5 |  | 0.0002 |
| ApoB | 129 | 59.7 |  | 54 | 75.0 |  | 75 | 52.8 |  | 0.001 |

ApoB, apolipoprotein B; C, cholesterol; HDL, high-density lipoprotein; LLT, lipid-lowering therapy; LDL, low-density lipoprotein. ^*^ Differences between LLT and no LLT determined by Chi-Square test. Two patients with missing information about LLT excluded.

**Suppl. Table 8.** Agreement in target attainment across recommended lipid biomarkers stratified by lipid-lowering therapy – LDL-cholesterol estimated based on Friedewald.

| Target attainment | LLT  (n=72) | No LLT  (n= 142) | *P*-diff^*^ |
| --- | --- | --- | --- |
| None | 18 (25.0) | 66 (46.5) | 0.002 |
| All three | 15 (20.8) | 9 (6.3) | 0.001 |
| Only LDL-C | 0 | 0 | 0 |
| Only non-HDL-C | 0 | 1 (0.7) | 1 |
| Only ApoB | 19 (26.4) | 44 (31.0) | 0.485 |
| LDL-C and non-HDL-C | 0 | 0 | 0 |
| LDL-C and ApoB | 1 (1.4) | 0 | 1 |
| Non-HDL-C and ApoB | 19 (26.4) | 22 (15.5) | 0.056 |

Data provided are frequency (percentage), n=214. Two patients with missing information about LLT excluded. ApoB, apolipoprotein B; C, cholesterol; high-density lipoprotein; LLT, lipid-lowering therapy; LDL, low-density lipoprotein. ^*^ Differences between LLT and no LLT determined by Chi-Square test.

**Suppl. Table 9.** Agreement in target attainment across recommended lipid biomarkers stratified by lipid-lowering therapy – LDL-cholesterol estimated based on Sampson.

| Target attainment | LLT  (n=72) | No LLT  (n= 142) | *P*-diff^*^ |
| --- | --- | --- | --- |
| None | 18 (25.0) | 66 (46.5) | 0.002 |
| All three | 14 (19.4) | 9 (6.3) | 0.003 |
| Only LDL-C | 0 | 0 | 0 |
| Only non-HDL-C | 0 | 1 (0.7) | 1 |
| Only ApoB | 20 (27.8) | 44 (31.0) | 0.628 |
| LDL-C and non-HDL-C | 0 | 0 | 0 |
| LDL-C and ApoB | 0 | 0 | 0 |
| Non-HDL-C and ApoB | 20 (27.8) | 22 (15.5) | 0.033 |

Data provided are frequency (percentage), n=214. Two patients with missing Information about LLT excluded. ApoB, apolipoprotein B; C, cholesterol; high-density lipoprotein; LLT, lipid-lowering therapy; LDL, low-density lipoprotein. ^*^ Differences between LLT and no LLT determined by Chi-Square test.

**Suppl. Table 10.** Agreement in target attainment across recommended lipid biomarkers stratified by lipid-lowering therapy – directly measured LDL-cholesterol.

| Target attainment | LLT  (n=72) | No LLT  (n= 142) | *P*-diff^*^ |
| --- | --- | --- | --- |
| None | 18 (25.0) | 66 (46.5) | 0.002 |
| All three | 9 (12.5) | 8 (5.6) | 0.079 |
| Only LDL-C | 0 | 0 | 0 |
| Only non-HDL-C | 0 | 1 (0.7) | 1 |
| Only ApoB | 20 (27.8) | 44 (31.0) | 0.628 |
| LDL-C and non-HDL-C | 0 | 0 | 0 |
| LDL-C and ApoB | 0 | 0 | 0 |
| Non-HDL-C and ApoB | 25 (34.7) | 23 (16.2) | 0.002 |

Data provided are frequency (percentage), n=214. Two patients with missing information about LLT excluded. ApoB, apolipoprotein B; C, cholesterol; high-density lipoprotein; LLT, lipid-lowering therapy; LDL, low-density lipoprotein. ^*^ Differences between LLT and no LLT determined by Chi-Square test.

**Suppl. Table 11**. The proportion of patients that reach the ESC lipid targets overall and stratified by sex.

|  | All  (n=216) | |  | Females  (n=85) | |  | Males  (n=131) | |  | *P*-diff* |
| --- | --- | --- | --- | --- | --- | --- | --- | --- | --- | --- |
| Target attainment | n | % |  | n | % |  | n | % |  |  |
| LDL-C, Friedewald^*^ | 25 | 11.6 |  | 7 | 8.2 |  | 18 | 13.7 |  | 0.22 |
| LDL-C, Sampson^*^ | 23 | 10.7 |  | 5 | 5.9 |  | 18 | 13.7 |  | 0.07 |
| LDL-C, measured^*^ | 17 | 7.9 |  | 4 | 4.7 |  | 13 | 9.9 |  | 0.20^**^ |
| Non-HDL-C cholesterol | 66 | 30.6 |  | 24 | 28.2 |  | 42 | 32.1 |  | 0.55 |
| ApoB | 129 | 59.7 |  | 52 | 61.2 |  | 77 | 58.8 |  | 0.73 |

ApoB, apolipoprotein B; C, cholesterol; HDL, high-density lipoprotein; LDL, low-density lipoprotein. ^*^ Differences between females and males determined by Chi-Square test

^**^ Determined by Fisher Exact test

**Suppl. Table 12**. The proportion of patients on lipid-lowering therapy stratified by ASCVD risk and sex.

|  | Females^£^  (n=84) | |  | Males^£^  (n=130) | | *P*-diff^*^ | |
| --- | --- | --- | --- | --- | --- | --- | --- |
| ASCVD risk | n | % |  | n | % |  |  |
| Moderate CV risk | 0 | 0 |  | 5 | 26.3 |  | 0.13^**^ |
| High CV risk | 15^£^ | 22.1 |  | 32^£^ | 34.8 |  | 0.08 |
| Very high CV risk | 3 | 50.0 |  | 17 | 89.5 |  | 0.069^**^ |
| Overall | 18 | 21.4 |  | 54 | 41.5 |  | 0.002 |

ASCVD, atherosclerotic cardiovascular disease; CV, cardiovascular.

^£^ Information on LLT missing in one patient ^*^ Differences between

females and males determined by Chi-Square test ^**^ Determined by

Fisher Exact test. See Supplemental Table 1 for definition of ASCVD risk.

**Suppl. Table 13.** Associations between age and sex and prescription of lipid-lowering medication (n=216).

|  | **Model 1** | | |  | **Model 2** | | |  | **Model 3** | | |
| --- | --- | --- | --- | --- | --- | --- | --- | --- | --- | --- | --- |
| Characteristic | OR | 95% CI | *P* |  | OR | 95% CI | *P* |  | OR | 95% CI | *P* |
| **Female sex** | 0.38 | (0.21, 0.72) | 0.0027 |  | 0.35 | (0.16, 0.77) | 0.009 |  | 0.37 | (0.17, 0.83) | 0.02 |
| Age, yrs | - | - | - |  | 1.10 | (1.07, 1.13) | <0.0001 |  | 1.09 | (1.06, 1.12) | <0.0001 |
| Diabetes duration, yrs | - | - | - |  | 1.04 | (1.00, 1.08) | 0.03 |  | 1.04 | (1.00, 1.08) | 0.08 |
| HbA1c, % |  |  |  |  | 1.23 | (0.89, 1.71) | 0.21 |  | 1.14 | (0.82, 1.58) | 0.43 |
| ASCVD risk | - | - | - |  | - | - | - |  |  |  |  |
| Moderate | - | - | - |  | - | - | - |  | ref |  |  |
| High | - | - | - |  | - | - | - |  | 1.09 | (0.30, 3.95) | 0.90 |
| Very high | - | - | - |  | - | - | - |  | 5.06 | (0.86, 29.86) | 0.07 |
|  |  |  |  |  |  |  |  |  |  |  |  |
| **Above median age** | 10.00 | (4.92, 20.34) | <0.0001 |  | 8.61 | (4.08, 18.19) | <0.0001 |  | 7.50 | (3.53, 15.96) | <0.0001 |
| Female sex | - | - | - |  | 0.33 | (0.16, 0.70) | 0.004 |  | 0.36 | (0.17, 0.78) | 0.009 |
| Diabetes duration, yrs | - | - | - |  | 1.05 | (1.02, 1.09) | 0.002 |  | 1.05 | (1.01, 1.09) | 0.01 |
| HbA1c, % |  |  |  |  | 1.14 | (0.83, 1.57) | 0.41 |  | 1.07 | (0.78, 1.46) | 0.69 |
| ASCVD risk | - | - | - | - | - | - | - |  |  |  |  |
| Moderate | - | - | - | - | - | - | - |  | ref |  |  |
| High | - | - | - | - | - | - | - |  | 0.91 | (0.26, 3.13) | 0.88 |
| Very high | - | - | - | - | - | - | - |  | 5.03 | (0.93, 27.28) | 0.06 |

Data are odds ratios (OR) with 95% confidence interval (CI) in brackets from logistic regression analysis. ASCVD, atherosclerotic cardiovascular disease;

HbA1c, hemoglobin A1c.

**Suppl. Table 14.** The proportion of patients on lipid-lowering therapy stratified by ASCVD risk and median age.

|  | ≤43.6 yrs  (n=106)^£^ | |  | >43.6 yrs  (n=108) | | *P*-diff^*^ | |  |
| --- | --- | --- | --- | --- | --- | --- | --- | --- |
| ASCVD risk | n | % |  | n | % |  |  |  |
| Moderate | 2 | 10.0 |  | 3 | 33.3 |  | 0.29^**^ |  |
| High | 10^£^ | 12.2 |  | 37 | 47.4 |  | <0.00001 | |
| Very high | 0 | 0 |  | 20 | 95.2 |  | 0.0004^**^ |  |
| Total | 12 | 11.3 |  | 60 | 55.6 |  | <0.00001 |  |

ASCVD, atherosclerotic cardiovascular disease; CV, cardiovascular. ^£^ Information on LLT missing in two patients ^*^ Determined by Chi-Square test ^**^ Determined by Fisher Exact test. See Supplemental Table 1 for definition of ASCVD risk.

**Suppl. Table 15.** The distribution of ASCVD risk and the proportion of patients that reach ESC lipid targets stratified by median age.

|  | ≤43.6 yrs (n=108) | |  | >43.6 yrs  (n=108) | | *P*-diff^*^ | |
| --- | --- | --- | --- | --- | --- | --- | --- |
|  | n | % |  | n | % |  |  |
| ASCVD risk |  |  |  |  |  |  |  |
| Moderate | 20 | 18.5 |  | 9 | 8.3 |  | 0.03 |
| High | 84 | 77.8 |  | 78 | 72.2 |  | 0.35 |
| Very high | 4 | 3.7 |  | 21 | 19.4 |  | 0.0004^**^ |
| Lipid target attainment |  |  |  |  |  |  |  |
| LDL-C, Friedewald | 10 | 9.3 |  | 15 | 13.9 |  | 0.29 |
| LDL-C, Sampson | 9 | 8.3 |  | 14 | 13.0 |  | 0.27 |
| LDL-C, measured | 7 | 6.5 |  | 10 | 9.3 |  | 0.45 |
| Non-HDL-cholesterol | 34 | 31.5 |  | 32 | 29.6 |  | 0.77 |
| ApoB | 61 | 56.5 |  | 68 | 63.0 |  | 0.33 |

ApoB, apolipoprotein B; ASCVD, atherosclerotic cardiovascular disease C, cholesterol; CV,

cardiovascular; HDL, high-density lipoprotein; LDL, low-density lipoprotein. ^*^ Differences determined by Chi-Square test ^**^ Determined by Fisher Exact test. See Supplemental Table 1 for definition of ASCVD risk.

**Suppl. Table 16.** Basic characteristics of included and excluded patients.

|  | Included patients  (n=216) | | Excluded patients  (n=106) | |  | |
| --- | --- | --- | --- | --- | --- | --- |
| Characteristics | n | median (IQR) or % | n | median (IQR) or % | *P*-value^e^ |  |
| Females | 85 | 39.4 | 33 | 31.1 | 0.15 |  |
| Age, yrs | 216 | 43.6 (31.9-58.2) | 106 | 40.5 (28.7-56.2) | 0.21 |  |
| Years since diagnosis, yrs | 216 | 16 (10-25) | 103 | 19 (12-28) | 0.029 |  |
| HbA1c, % | 216 | 7.3 (6.8-8.0) | 101 | 7.0 (6.4-7.8) | 0.020 |  |
| HbA1c, mmol/mol | 216 | 56 (51-64) | 101 | 53 (46-62) | 0.020 |  |
| BMI, kg/m^2^ | 216 | 25.2 (23.0-27.9) | 99 | 23.9 (21.9-26.7) | 0.006 |  |
| Systolic BP, mmHg | 215 | 128 (118-138) | 94 | 124 (116-137) | 0.10 |  |
| Diastolic BP, mmHg | 215 | 78 (73-84) | 94 | 78 (73-83) | 0.65 |  |
| Current smoker | 41 | 19.0 | 22^c^ | 22.2 | 0.50 |  |
| Lipid levels, mmol/L |  |  |  |  |  |  |
| Triglycerides | 216 | 0.9 (0.7-1.1) | 96 | 0.9 (0.7-1.3) | 0.84 |  |
| HDL-C | 216 | 1.5 (1.3-1.8) | 96 | 1.5 (1.3-1.9) | 0.85 |  |
| LDL-C^a^ | 216 | 2.7 (2.1-3.3) | 96 | 2.2 (1.6-2.8) | <0.0001 |  |
| Non-HDL-C | 216 | 3.1 (2.5-3.8) | 96 | 2.6 (2.0-3.3) | <0.0001 |  |
| Lipid-lowering medication | 72^b^ | 33.6 | 39^d^ | 39.8 | 0.29 |  |

BP, blood pressure; C, cholesterol; HDL, high-density lipoprotein; HbA1c, hemoglobin A1c; LDL,

low-density lipoprotein. ^a^ LDL-cholesterol estimated with the Friedewald formula ^b^ Information missing

in two patients ^c^ Information missing in seven patients ^d^ Information missing in eight patients ^e^ Based on

Chi-Square or Wilcoxon rank-sum test.
